# Drought resistance evaluation of sugar beet germplasms by response of phenotypic indicators

**DOI:** 10.1080/15592324.2023.2192570

**Published:** 2023-03-26

**Authors:** Wenbo Tan, Wangsheng Li, Jiajia Li, Dali Liu, Wang Xing

**Affiliations:** a National Beet Medium-term Gene Bank, Heilongjiang University, Harbin, P. R. China; b Key Laboratory of Sugar Beet Genetics and Breeding, College of Advanced Agriculture and Ecological Environment, Heilongjiang University, Harbin, P. R. China

**Keywords:** sugar beet germplasms, drought tolerance evaluation, phenotypic indicators, response

## Abstract

Sugar beet is a main sugar crop worldwide that often faces drought stress. The identification of drought tolerance of sugar beet germplasms is beneficial for breeding, but the research about it has been rarely reported. In this study, the drought tolerance of germplasms 92005-1, 94002-2 and 92021-1-1 was tested under simulated conditions. Seven days and 9% PEG treatment were the optimal conditions for evaluation, under which more phenotypic indicators showed significant difference in drought tolerance coefficient. The objective weighting and membership function method were established for evaluating the drought tolerance of different sugar beet germplasms. Drought stress decreased the biomass of leaves and roots of sugar beet germplasms. The drought-sensitive germplasm responded faster for leaf weight, root weight, plant height and root length. These indicators declined more significantly under long-term and severe stress. Increasing the root–shoot ratio and proline content were universal strategies of sugar beet germplasms to overcome drought stress. The drought-tolerant germplasms held higher peroxidase activity and better ability to scavenge reactive oxygen for preventing the damage.

## 1. Introduction

Sugar beet (*Beta vulgaris* L.) is a biennial herbaceous plant and an important sugar crop, which is tolerant to low temperature and salinity.^1^ Providing people with sugar needs consumption, it is often planted in semiarid region worldwide. The effect of drought on crops is a global concern, especially in areas with low precipitation.^2^ Selection for drought tolerance is meaningful to reduce the effects of water stress on crop yield.^3^ Investigation of biochemical and physiological response mechanisms under drought stress conditions may help provide a theoretical basis for selection and breeding of drought-resistant varieties of sugar beet, as well as the screening of drought tolerance indicators.

The moisture content in soil affects the morphological characteristics and physiological properties of plant.^4^ Investigation of drought tolerance at the seedling stage which has been widely adopted in recent years. Compared with traditional method, it holds the advantages of short test cycle, considerable capacity and high reproducibility.^5^ Much research about screening phenotypic indicators for drought tolerance has been carried out worldwide.^6,7^ The results showed that the uptake efficiency of soil water was positively correlated with root mass and root length of maize.^8^ Spike length and grain number or weight could be considered as intuitive indicators of drought tolerance of barley.^9^ Wheat demonstrated strong drought tolerance with better antioxidant capacity.^10^ The evaluation of drought tolerance of sugar beet was accomplished using complicated indices such as mean productivity (MP), geometric mean (GMP) and harmonic mean (HM). It usually lasts for 2 y in cropland, which is complex and low throughput.^11^ The drought resistance coefficient is more effective for selecting better genotypes with respect to tolerant, because a single index or comprehensive analysis of different traits has shortcomings in identifying drought resistance.^12^

In this study, drought stress was simulated on different germplasms that were selected from National Beet Medium-term Gene Bank according to the different growth vigor in arid field. By analyzing the indicators of sugar beet germplasms under different drought stress statistically, the suitable conditions for drought tolerance evaluation were determined. The response of phenotypic indicators was investigated, then they were used in calculation models for drought tolerance evaluation. The results provide the systematic methods which could be considered for the screening of drought-resistant sugar beet germplasms.

## 2. Materials and methods

### 2.1. Experimental treatment

The germplasms of sugar beet 92005-1, 94002-2 and 92021-1-1 were multigerm, fertile and diploid. Full seeds of uniform size were selected from each germplasm. They were soaked in 75% alcohol for 1 min in gauze, and then rinsed under running water. The seeds were soaked in 2% thiram solution overnight and sown uniformly in pots with vermiculite, then sprayed with water every day. After two cotyledons showed growth, 48 seedlings of each germplasm were selected and planted in 24-well pots with Hoagland solution.^13^ The seedlings were cultivated in phytotron under 25°C and 50% RH. The solution was changed every 7 d, and the sugar beet seedlings were subjected to drought stress after two true leaves were observed. The PEG-6000 concentration was set at 3%, 6%, and 9%. The control group was cultivated without PEG treatment. Each treatment was replicated three times.

### 2.2. Measurement of indicators

The root length and plant height were measured by ruler. The weight of different organs was measured by electronic balance. Superoxide dismutase was assayed using nitroblue tetrazolium method.^14^ Peroxidase was determined using guaiacol method.^15^ Soluble sugar, malondialdehyde and proline were assayed using anthrone colorimetry, thiobarbituric acid and acid ninhydrin methods, respectively.^16^ Soluble protein was assayed using bicinchoninic acid kit according to protocol of manufacturer. All indicators were tested three times.

### 2.3. Data processing and analysis

The coefficient of variation (CV) denotes the ratio of standard deviation to average of indicators. It can eliminate the effect of different units and means on the comparison of the degree of variation of two or more samples. The membership function value was calculated using formula 1, where *i* denotes germplasm and *j* denotes indicator. X_*j*_ max and X_*j*_ min indicate the maximum and minimum measurement of each indicator.

(1) $$U_{ij}=\left(X_{ij}-X_{j\;min}\right)/\left(X_{j\;max}-X_{j\;min}\right)$$

The relative drought tolerance coefficient I_*j*_ was calculated using formula 2, where C_*j*_ is the value of indicator in the control group and S_*j*_ is the value of indicator measured under drought stress treatment.

(2) $$I_{j}=C_{j}/S_{j}$$

(3) $$W_{j}=I_{j}/\Sigma I_{j}$$

(4) $$D=\Sigma \left(U_{ij}\times W_{j}\right)$$

The weight W_*j*_ was calculated by the objective weighting method using formula 3. Finally, the integrated assessment value was calculated using formula 4.

## 3. Results

### 3.1. Drought tolerance coefficients of indicators of germplasms subjected to treatment for 2 d under different levels of drought stress

The drought tolerance coefficient of indicators was analyzed by subjecting sugar beet germplasms to different concentrations of PEG for 2 d (Table S1). After subjected to 2 d of PEG stress, no indicators exhibited significant differences with control under 3% PEG treatment. Four and eight indicators varied dramatically under 6% and 9% PEG treatment, respectively. The leaf fresh weight, leaf dry weight, leaf saturated dry weight, and proline content were significantly different under both 6% and 9% PEG treatments. The root fresh weight, root dry weight, relative leaf water content and peroxidase activity showed difference uniquely under 9% PEG treatment. The results indicated that 9% PEG concentration led to a substantially different performance in drought tolerance among the three sugar beet germplasms under short-term treatment.

### 3.2. Drought tolerance coefficients of indicators of germplasms subjected to treatment for 7 d under different levels of drought stress

The drought tolerance coefficient of each indicator in three sugar beet germplasm resources was analyzed at different PEG concentrations (Table S2). The values of coefficient of root length, soluble protein content, soluble sugar content, superoxide dismutase activity, peroxidase activity, and malondialdehyde content showed no significant differences with any concentration. The coefficient of most indicators such as root length and soluble protein varied acutely under 3%, 6% and 9% treatments, which indicated that the effects of drought stress on the three sugar beet germplasms differed significantly. The coefficient of nine indicators, such as plant height, relative leaf water content, and root–shoot ratio were significantly different from control under 9% treatment. Thus, 7 d of long-term drought stress and 9% PEG concentration are the optimum conditions for drought tolerance test of sugar beet germplasms.

### 3.3. Evaluation of drought tolerance by indicators

The coefficient of indicators of each germplasm were analyzed under 7 d of long-term drought stress at 9% PEG concentration (Table 1). Among germplasms, almost all CV of coefficient of indicators were greater than 20%, indicating the selected indicators were suitable for evaluating drought tolerance of the germplasms. For different indicators, the order of coefficient among germplasms was randomized. Thus, the drought-resistant capacity of sugar beet germplasms could not be evaluated by using a single indicator, as the evaluation results would be inaccurate. Multiple indicators should be used for an effective, objective, and a comprehensive evaluation.

**Table 1.** Drought tolerance coefficients of indicators of different germplasms.

	RL	PH	RFW	LFW	LSFW	LDW	RDW	RLW	RSR	SP	Pro	SS	SOD	POD	MDA
92005-1	0.801	0.403	0.135	0.057	0.077	0.164	0.239	0.688	2.370	2.261	2.318	0.950	0.600	1.242	1.821
94002-2	0.437	0.445	0.063	0.029	0.037	0.084	0.075	0.731	2.329	0.441	2.356	0.311	0.947	0.725	0.875
92021-1-1	0.826	0.650	0.237	0.115	0.133	0.218	0.277	0.578	2.035	2.040	2.320	1.241	1.025	2.007	2.152
Average	0.688	0.499	0.145	0.067	0.082	0.155	0.197	0.666	2.245	1.581	2.331	0.834	0.857	1.325	1.616
CV (%)	25803	21.574	49.149	53.880	48.116	35.423	44.365	9.682	66.293	51.301	0.738	46.583	21.532	39.741	33.487

### 3.4. Comprehensive evaluation of drought tolerance by membership function and weight

Various indicators for subjecting the germplasms to 7 d of stress at 9% PEG concentration were selected via analysis of significant differences to eliminate the one-sidedness of individual indicator analysis. The root length, plant height, leaf fresh weight, root fresh weight, leaf dry weight, root dry weight, relative leaf water content, root–shoot ratio, leaf-saturated fresh weight, proline content, peroxidase activity, superoxidase activity, malondialdehyde content, soluble sugar content, and soluble protein content under conditions of drought stress were analyzed to comprehensively evaluate the seedling drought tolerance of the three sugar beet germplasm resources. The *D*-values were based on the membership function values and objective weights, and the drought tolerance of each germplasm was estimated in the order 92021-1-1 (0.791) > 92005-1 (0.613) > 94002-2 (0.226) (Table 2).

**Table 2.** Comprehensive evaluation of drought tolerance in the three sugar beet germplasms.

Indicators	92005-1		94002-2		92021-1-1	
	U_*ij*_	W_*j*_	U_*ij*_	W_*j*_	U_*ij*_	W_*j*_
RL	0.934	0.059	0.000	0.059	1.000	0.059
PH	0.000	0.036	0.172	0.036	1.000	0.036
RFW	0.411	0.024	0.000	0.024	1.000	0.024
LFW	0.324	0.012	0.000	0.012	1.000	0.012
LSFW	0.416	0.013	0.000	0.013	1.000	0.013
LDW	0.597	0.018	0.000	0.018	1.000	0.018
RDW	0.813	0.029	0.000	0.029	1.000	0.029
RLW	0.719	0.021	1.000	0.021	0.000	0.021
RSR	1.000	0.049	0.876	0.049	0.000	0.049
SP	1.000	0.269	0.000	0.269	0.879	0.269
Pro	0.000	0.006	1.000	0.006	0.038	0.006
SS	0.687	0.129	0.000	0.129	1.000	0.129
SOD	0.000	0.061	0.816	0.061	1.000	0.061
POD	0.404	0.174	0.000	0.174	1.000	0.174
MDA	0.124	0.100	1.000	0.100	0.000	0.100
*D*-value	0.613		0.226		0.791	

### 3.5. The changes in various indicators of sugar beet germplasms at different drought tolerance levels

#### 3.5.1. Effects of drought stress on leaf dry weight, leaf fresh weight, and leaf-saturated fresh weight of sugar beet germplasms

**Figure 1.**
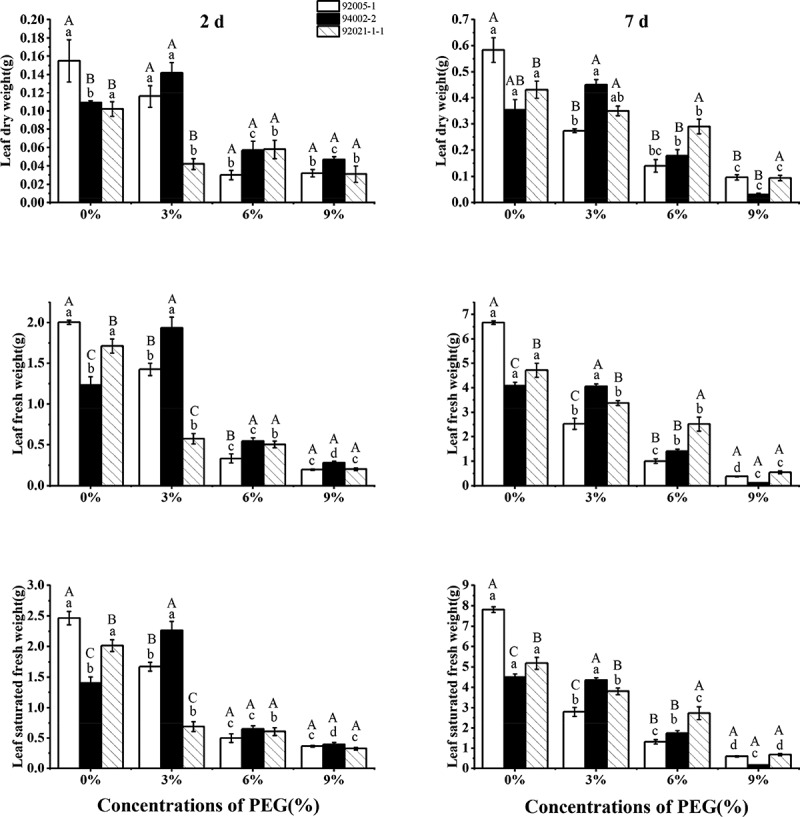
Variation of leaf dry weight, fresh weight, and saturated fresh weight of sugar beet germplasms. Different lowercase letters indicate that the same germplasm shows a significant difference under different PEG concentrations (*P* < 0.05). Capital letters indicate a significant difference between different germplasms under conditions of subjection to the same PEG concentration (*P* < 0.05). .

As shown in Figure 1, the leaf fresh weight, leaf dry weight, and leaf saturated fresh weight of the germplasms with strong drought tolerance (92021-1-1 and 92005-1, D > 0.5) decreased with increasing levels of drought stress in short and long term. The indicators of the germplasm with weak drought tolerance (94002-2, *D* < 0.5) increased at 3% PEG concentration and then decreased with increasing drought stress in short term. For 7 d treatment, the indicators of all germplasms declined with increasing stress. Finally, the leaf dry weight and leaf fresh weight of 94002-2 decreased by 91.53% and 97.14%, respectively, which were much greater than strong drought-tolerant germplasms.

#### 3.5.2. Effects of drought stress on root dry weight and root fresh weight of sugar beet germplasms

**Figure 2.**
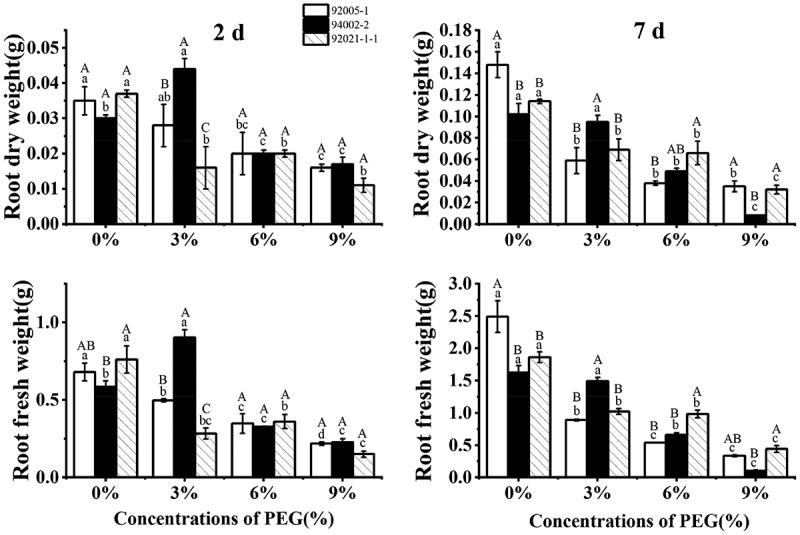
Variation of root dry weight and root fresh weight of sugar beet germplasms.

As depicted in Figure 2, the root dry weight and root fresh weight of all germplasms showed decrease with increasing drought stress, except for the increase of 94002-2 at the beginning of treatment with low PEG concentration. The final reduction rate of root dry weight (91.52%) and fresh weight (93.69%) of weak drought-tolerant germplasm was also more significant than the others.

#### 3.5.3. Effects of drought stress on plant height and root length of sugar beet germplasms

**Figure 3.**
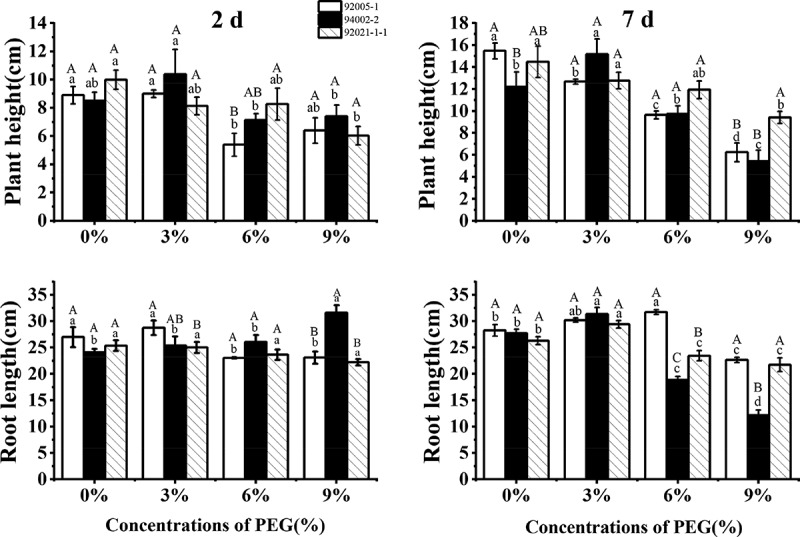
Variation of plant height and root length of sugar beet germplasms.

As shown in Figure 3, the plant height of all germplasms showed decreasing trend with increasing drought stress, except for the increase of poor drought-tolerant germplasm at 3% PEG concentration with short- and long-term treatment. The root length of 94002-2 kept increasing with increasing drought stress through short-term treatment process. With long-term treatment, the root length of all germplasms increased under slight drought stress and declined under high PEG concentration finally.

#### 3.5.4. Effects of drought stress on relative leaf water content and root–shoot ratio of sugar beet germplasms

**Figure 4.**
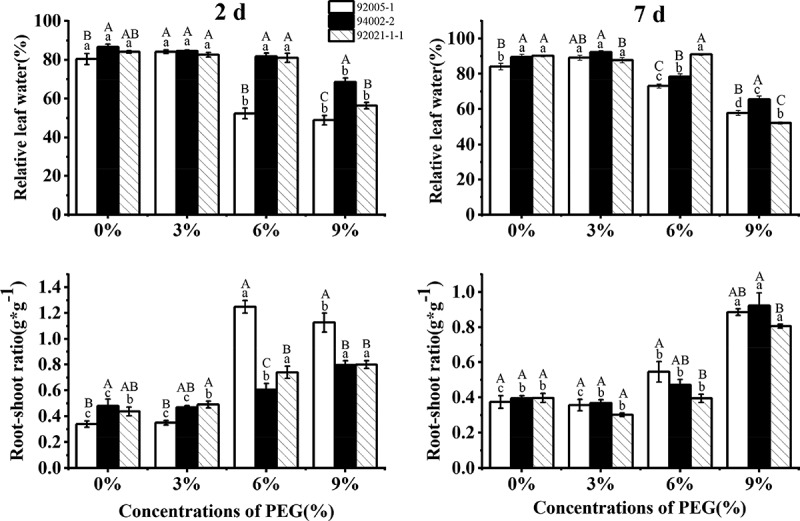
Variation of relative leaf water and root–shoot ratio of sugar beet germplasms.

As shown in Figure 4, the relative leaf water of three germplasms almost did not change under low PEG concentration, and then decreased significantly with subjection to 9% PEG treatment for 7 d. The root–shoot ratio of sugar beet germplasms was almost changeless with mild stress, and then increased by 137% (92005-1), 133% (94002-2) and 103% (92021-1-1) finally.

#### 3.5.5. Effects of drought stress on osmoregulatory substances of sugar beet germplasms

**Figure 5.**
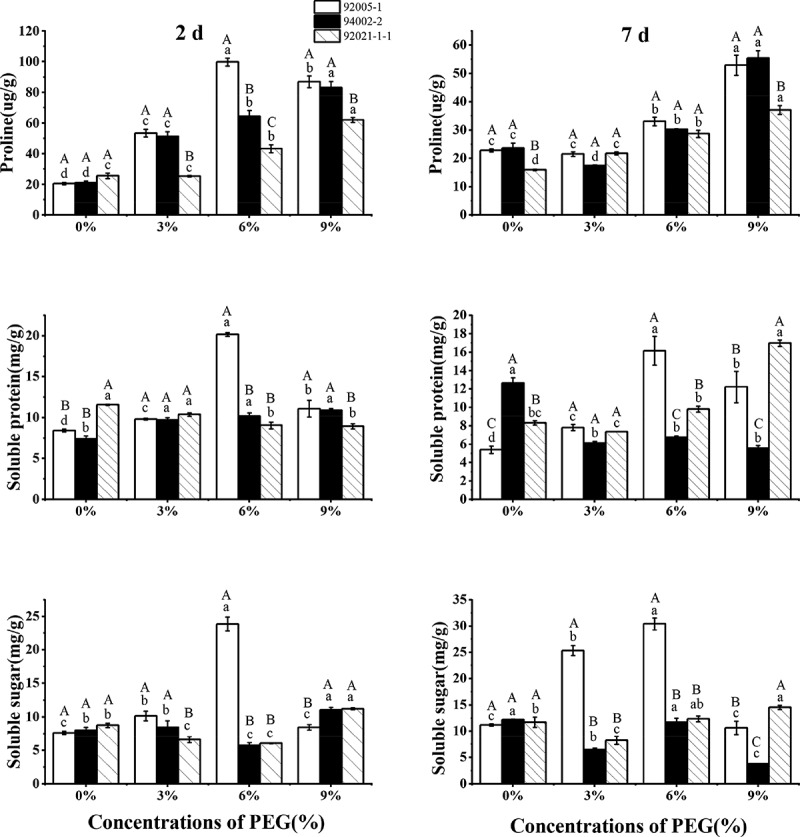
Variation of osmoregulatory substances of sugar beet germplasms.

The proline content of three sugar beet germplasms increased significantly under short- and long-term drought treatment with increasing drought stress, which increased by 131% (92005-1), 136% (94002-2) and 132% (92021-1-1) finally. The soluble protein content of germplasms showed obvious changes under drought stress and the strong drought-tolerani germplasms increased significantly under long-term and severe stress. However, the soluble protein content of weak drought-tolerant germplasm (94002-2) decreased significantly (56%) under the same stress condition. The soluble sugar content of germplasms showed a tendency to increase under short-term stress, but the germplasm 94002-2 showed a sharp decline (69%) with severe drought stress (Figure 5).

#### 3.5.6. Effects of drought stress on antioxidant enzyme activity of sugar beet germplasms

**Figure 6.**
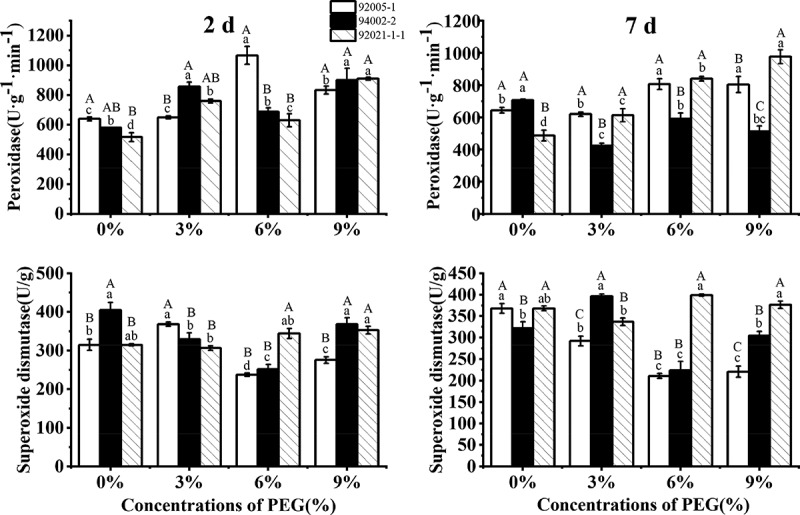
Variation of antioxidant enzyme activities of sugar beet germplasms.

Peroxidase activity of all sugar beet germplasms increased significantly under short-term treatment with increasing drought stress. Under long-term and severe stress, the strong drought-tolerant germplasms responded by increasing peroxidase activity. However, the weak drought-tolerant germplasm (94002-2) decreased by 27% significantly. The superoxide dismutase activity of sugar beet germplasms almost did not change markedly except for 92005-1 (Figure 6).

#### 3.5.7. Effects of drought stress on malondialdehyde content of sugar beet germplasms

**Figure 7.**
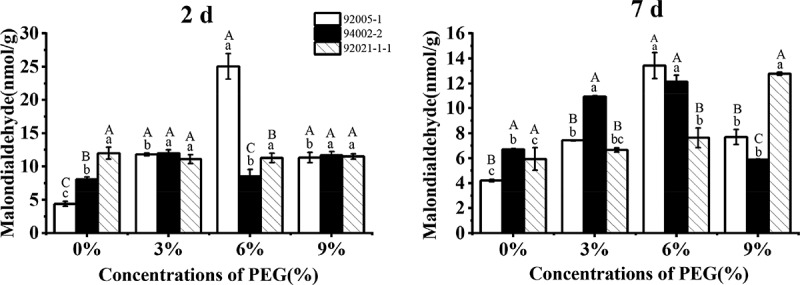
Variation of malondialdehyde content of sugar beet germplasms.

The drought stress promoted the malondialdehyde content of sugar beet germplasms, but the germplasm with the strongest drought tolerance (92021-1-1) did not respond by malondialdehyde under short-term drought treatment. Under long-term drought stress, the malondialdehyde content of germplasm 92021-1-1 increased significantly by 115%. The others showed a marked decline under long-term and high PEG concentration treatment (Figure 7).

## 4. Discussion

Drought tolerance evaluation of crops is often carried out using physiological and biochemical indicators, such as plant height and superoxide dismutase.^17^ If throughput and cost of time and operation are not limited, some comprehensive indices are also used for evaluation in field. Geometric mean productivity, percentage of yield reduction and yield index has been employed effectively in multi-plot and long-term evaluation.^18^ The evaluation of crop drought tolerance relies not only on the selection of suitable indicators but also relies on the selection of suitable methods.^19^ Much research uses equally weighted evaluation and ignore the importance of different indicators. In the present study, the objective weighting method was used to calculate the weight of each indicator, and the drought tolerance of each indicator was calculated by membership function to eliminate the differences generated by using different units of indicators. It makes the results of evaluation more reliable, which are consistent with the drought tolerance performance of germplasms in field investigation.

Plant leaves play a vital role in plant life activities, which are pioneers for response to drought stress. Drought stress reduces water uptake and triggers the closure of the stomata. The decrease of water and carbon dioxide content disrupts photosynthesis, so the organic matter of leaves decline.^20^ The leaf fresh weight, leaf dry weight and leaf saturated fresh weight of germplasms declined sharply under long-time and severe drought conditions. But the weak drought-tolerant germplasm seemed to be more positive under mild stress conditions and showed an increase.

The root system is the first to sense drought. Under slight drought stress, plant organic matter is preferentially transported to the root system. The development and activities of root system will promote under endurable stress. It helps plants overcome severe drought stress.^21,22^ The results of the present study showed that the root length of all sugar beet germplasms increased under 7 d slight drought stress to uptake limited water. The germplasm with weak drought tolerance was more sensitive, of which the root length kept increasing with short-term treatment. The germplasm 94002-2 also adopted the same strategies for root dry weight and fresh weight, but the decline degree of root indicators was inversely proportional to the strength of drought tolerance finally. The germplasms with strong drought tolerance could ensure the accumulation of dry matter in roots under severe drought conditions. All sugar beet germplasms increased root–shoot ratio to improve the efficiency of water uptake with drought treatment.

The lack of availability of water could promote the production of reactive oxygen, which causes damage to membrane components, proteins, and DNA.^23,24^ The osmoregulatory substances and antioxidant protective enzymes are defense system of plants against drought stress. Peroxidase activity of strong drought-tolerant germplasms increased significantly, but the weak showed a marked decline. Peroxidase activity of different sugar beet was variable according to the drought resistance level, while the superoxide dismutase activity was not sensitive.

When plants are threatened by drought, they will increase the content of soluble substances to meet the normal water demand.^25^ Proline not only improves the osmoregulatory capacity of plants but also could scavenge hydroxyl radicals.^26^ Soluble protein and sugar were transferred from the roots for growth and development of leaves under drought stress.^27^ Increasing proline content was a universal strategy of different sugar beet germplasms to overcome drought stress. For soluble protein and sugar, the weak drought-tolerant germplasm was deficient and showed a sharp decrease under drought stress.

Maintaining the integrity and stability of the cell membrane is one of the important factors for drought tolerance in plants.^28^ Free radicals act on lipids to undergo peroxidation reactions and the end-product is malondialdehyde. High malondialdehyde content indicates a high degree of peroxidation in the cell membrane which is severely damaged.^29^ The strong drought-tolerant germplasm had better ability to scavenge reactive oxygen under short-term stress, and the cell membrane was not damaged. After reaching the tolerance threshold, the reductive organic substances of cell membrane decreased, resulting in a decrease of malondialdehyde content. The increasing malondialdehyde content of the strong drought-resistant germplasm proved that it still maintained life activities through free oxygen scavenging system within the tolerance range.

## Supplementary Material

Supplemental Material
