# Supplementary material for: Drought resistance evaluation of sugar beet germplasms by response of phenotypic indicators
Source: Plant Signal Behav. 2023 Mar 26;18(1):2192570. doi: 10.1080/15592324.2023.2192570 (PMC10044154; doi:10.1080/15592324.2023.2192570)
Supplement: Supplementary file 1 [file KPSB_A_2192570_SM9102.odt]

Table S1. Analysis of drought tolerance coefficient of each indicator after 2 days of subjection to different PEG concentrations.

| Indicators | PEG Concentration (%) | Average | Min | Max | CV (%) |
| --- | --- | --- | --- | --- | --- |
| RL | 0 | 1.000 a | 1.000 | 1.000 | 0.000 |
| 3 | 1.036 a | 0.987 | 1.067 | 4.151 |
| 6 | 0.957 a | 0.857 | 1.083 | 12.017 |
| 9 | 1.015 a | 0.857 | 1.314 | 25.517 |
| PH | 0 | 1.000 ab | 1.000 | 1.000 | 0.000 |
| 3 | 1.015 a | 0.815 | 1.220 | 20.000 |
| 6 | 0.757 ab | 0.605 | 0.839 | 17.437 |
| 9 | 0.731 b | 0.604 | 0.871 | 18.331 |
| RFW | 0 | 1.000 a | 1.000 | 1.000 | 0.000 |
| 3 | 0.883 ab | 0.372 | 1.548 | 68.290 |
| 6 | 0.514 ab | 0.473 | 0.559 | 8.366 |
| 9 | 0.303 b | 0.197 | 0.389 | 32.013 |
| LFW | 0 | 1.000 a | 1.000 | 1.000 | 0.000 |
| 3 | 0.873 ab | 0.337 | 1.570 | 72.394 |
| 6 | 0.302 bc | 0.167 | 0.443 | 45.695 |
| 9 | 0.149 c | 0.097 | 0.230 | 47.651 |
| LSFW | 0 | 1.000 a | 1.000 | 1.000 | 0.000 |
| 3 | 0.877 ab | 0.342 | 1.611 | 74.914 |
| 6 | 0.324 bc | 0.203 | 0.467 | 41.049 |
| 9 | 0.198 c | 0.149 | 0.281 | 36.869 |
| LDW | 0 | 1.000 a | 1.000 | 1.000 | 0.000 |
| 3 | 0.821 ab | 0.407 | 1.304 | 55.177 |
| 6 | 0.426 bc | 0.194 | 0.564 | 47.418 |
| 9 | 0.315 c | 0.208 | 0.436 | 36.508 |
| RDW | 0 | 1.000 a | 1.000 | 1.000 | 0.000 |
| 3 | 0.896 ab | 0.436 | 1.462 | 58.147 |
| 6 | 0.596 ab | 0.555 | 0.670 | 10.738 |
| 9 | 0.440 b | 0.300 | 0.571 | 30.909 |
| RLW | 0 | 1.000 a | 1.000 | 1.000 | 0.000 |
| 3 | 1.002 a | 0.976 | 1.047 | 3.892 |
| 6 | 0.853 ab | 0.651 | 0.964 | 20.516 |
| 9 | 0.690 b | 0.608 | 0.792 | 13.623 |
| RSR | 0 | 1.000 a | 1.000 | 1.000 | 0.000 |
| 3 | 1.044 a | 0.975 | 1.125 | 7.280 |
| 6 | 2.214 a | 1.260 | 3.687 | 58.446 |
| 9 | 2.270 a | 1.658 | 3.324 | 40.352 |
| SP | 0 | 1.000 a | 1.000 | 1.000 | 0.000 |
| 3 | 1.123 a | 0.898 | 1.306 | 18.433 |
| 6 | 1.515 a | 0.781 | 2.393 | 53.861 |
| 9 | 1.186 a | 0.774 | 1.469 | 30.776 |
| Pro | 0 | 1.000 b | 1.000 | 1.000 | 0.000 |
| 3 | 2.015 ab | 0.985 | 2.612 | 44.467 |
| 6 | 3.219 a | 1.689 | 4.894 | 49.922 |
| 9 | 3.539 a | 2.416 | 4.239 | 27.748 |
| SS | 0 | 1.000 a | 1.000 | 1.000 | 0.000 |
| 3 | 1.048 a | 0.754 | 1.331 | 27.576 |
| 6 | 1.514 a | 0.691 | 3.132 | 92.602 |
| 9 | 1.258 a | 1.109 | 1.382 | 10.970 |
| SOD | 0 | 1.000 a | 1.000 | 1.000 | 0.000 |
| 3 | 0.984 a | 0.810 | 1.168 | 18.191 |
| 6 | 0.824 a | 0.623 | 1.095 | 29.612 |
| 9 | 0.968 a | 0.874 | 1.120 | 13.740 |
| POD | 0 | 1.000 b | 1.000 | 1.000 | 0.000 |
| 3 | 1.323 ab | 1.021 | 1.476 | 19.803 |
| 6 | 1.359 ab | 1.191 | 1.666 | 19.573 |
| 9 | 1.544 a | 1.299 | 1.771 | 15.350 |
| MDA | 0 | 1.000 a | 1.000 | 1.000 | 0.000 |
| 3 | 1.697 a | 0.926 | 2.684 | 52.976 |
| 6 | 2.564 a | 0.941 | 5.695 | 105.772 |
| 9 | 1.661 a | 0.959 | 2.579 | 50.030 |

Same letter indicates no significant difference (P > 0.05), and different letters indicate significant difference (P < 0.05). CV, coefficient of variation; RL, root length; PH, plant height; RFW, root fresh weight; LFW, leaf fresh weight; LSFW, leaf saturated fresh weight; LDW, leaf dry weight; RDW, root dry weight; RLW, relative leaf water; RSR, root-shoot ratio; SP, soluble protein; Pro, proline; SS, soluble sugar; SOD, superoxide dismutase; POD, peroxidase; MDA, malondialdehyde. The same below.

Table S2. Analysis of drought tolerance coefficient of each indicator after 7 days of subjection to different PEG concentrations.

| Indicators | PEG concentration    (%) | Average | Min | Max | CV (%) |
| --- | --- | --- | --- | --- | --- |
| RL | 0 | 1.000 a | 1.000 | 1.000 | 0.000 |
| 3 | 1.021 a | 0.815 | 1.130 | 17.532 |
| 6 | 0.898 a | 0.680 | 1.123 | 24.722 |
| 9 | 0.688 a | 0.437 | 0.826 | 31.686 |
| PH | 0 | 1.000 a | 1.000 | 1.000 | 0.000 |
| 3 | 0.981 a | 0.819 | 1.243 | 23.344 |
| 6 | 0.749 ab | 0.623 | 0.825 | 14.686 |
| 9 | 0.499 b | 0.403 | 0.650 | 26.453 |
| RFW | 0 | 1.000 a | 1.000 | 1.000 | 0.000 |
| 3 | 0.609 b | 0.357 | 0.920 | 46.962 |
| 6 | 0.384 bc | 0.216 | 0.528 | 40.885 |
| 9 | 0.145 c | 0.063 | 0.237 | 60.000 |
| LFW | 0 | 1.000 a | 1.000 | 1.000 | 0.000 |
| 3 | 0.697 a | 0.380 | 0.994 | 44.046 |
| 6 | 0.343 b | 0.151 | 0.533 | 55.685 |
| 9 | 0.067 b | 0.029 | 0.115 | 65.672 |
| LSFW | 0 | 1.000 a | 1.000 | 1.000 | 0.000 |
| 3 | 0.687 ab | 0.358 | 0.969 | 44.833 |
| 6 | 0.362 bc | 0.169 | 0.528 | 50.000 |
| 9 | 0.082 c | 0.037 | 0.133 | 58.537 |
| LDW | 0 | 1.000 a | 1.000 | 1.000 | 0.000 |
| 3 | 0.851 ab | 0.469 | 1.276 | 47.591 |
| 6 | 0.472 bc | 0.240 | 0.672 | 46.186 |
| 9 | 0.155 c | 0.084 | 0.218 | 43.226 |
| RDW | 0 | 1.000 a | 1.000 | 1.000 | 0.000 |
| 3 | 0.648 b | 0.402 | 0.938 | 41.821 |
| 6 | 0.438 bc | 0.260 | 0.574 | 36.758 |
| 9 | 0.197 c | 0.075 | 0.277 | 54.315 |
| RLW | 0 | 1.000 a | 1.000 | 1.000 | 0.000 |
| 3 | 1.022 a | 0.973 | 1.061 | 4.403 |
| 6 | 0.918 a | 0.869 | 1.009 | 8.606 |
| 9 | 0.666 b | 0.578 | 0.731 | 11.862 |
| RSR | 0 | 1.000 bc | 1.000 | 1.000 | 0.000 |
| 3 | 0.882 c | 0.762 | 0.955 | 11.905 |
| 6 | 1.215 b | 0.997 | 1.459 | 19.095 |
| 9 | 2.245 a | 2.035 | 2.370 | 8.151 |
| SP | 0 | 1.000 a | 1.000 | 1.000 | 0.000 |
| 3 | 0.938 a | 0.483 | 1.445 | 51.493 |
| 6 | 1.567 a | 0.534 | 2.988 | 81.174 |
| 9 | 1.581 a | 0.441 | 2.261 | 62.808 |
| Pro | 0 | 1.000 a | 1.000 | 1.000 | 0.000 |
| 3 | 1.020 b | 0.743 | 1.366 | 31.078 |
| 6 | 1.509 c | 1.288 | 1.788 | 16.899 |
| 9 | 2.331 c | 2.318 | 2.356 | 0.901 |
| SS | 0 | 1.000 a | 1.000 | 1.000 | 0.000 |
| 3 | 1.164 a | 0.530 | 2.260 | 81.873 |
| 6 | 1.575 a | 0.956 | 2.714 | 62.667 |
| 9 | 0.834 a | 0.311 | 1.241 | 57.074 |
| SOD | 0 | 1.000 a | 1.000 | 1.000 | 0.000 |
| 3 | 0.982 a | 0.795 | 1.231 | 22.912 |
| 6 | 0.784 a | 0.573 | 1.086 | 34.311 |
| 9 | 0.857 a | 0.600 | 1.025 | 26.371 |
| POD | 0 | 1.000 a | 1.000 | 1.000 | 0.000 |
| 3 | 0.941 a | 0.604 | 1.260 | 34.857 |
| 6 | 1.270 a | 0.836 | 1.726 | 35.039 |
| 9 | 1.325 a | 0.725 | 2.007 | 48.679 |
| MDA | 0 | 1.000 a | 1.000 | 1.000 | 0.000 |
| 3 | 1.501 a | 1.120 | 1.756 | 22.385 |
| 6 | 2.090 a | 1.285 | 3.177 | 46.746 |
| 9 | 1.616 a | 0.875 | 2.152 | 41.027 |
